# Retinal honeycomb appearance and its role in patients with X-linked retinoschisis

**DOI:** 10.1186/s12886-023-02835-2

**Published:** 2023-03-06

**Authors:** Jing Ma, Jing-Hua Liu, Song-Feng Li, Yan Ma, Guang-Da Deng, Liang Li, Ming-Zhen Yuan, Hai Lu

**Affiliations:** Beijing Tongren Hospital, Capital Medical University, Beijing Tongren Eye Center, Key Laboratory of Beijing Ophthalmology and Visual Science, Beijing, 100730 China

**Keywords:** Retinoschisis/congenital, Diagnostic imaging, Retina

## Abstract

**Background:**

To investigate the clinical characteristics of retinal honeycomb appearance in a large cohort of patients with X-linked retinoschisis (XLRS) and to determine whether it is associated with complications like retinal detachment (RD) and vitreous hemorrhage (VH).

**Methods:**

A retrospective observational case series. A chart review of medical records, wide-field fundus imaging, and optical coherence tomography (OCT) was performed on 78 patients (153 eyes) diagnosed with XLRS at Beijing Tongren eye center between Dec 2017 and Feb 2022. The chi-square test or Fisher exact test was performed on the 2 × 2 cross-tabulations of honeycomb appearance and other peripheral retinal findings and complications.

**Results:**

Thirty-eight patients (48.7%), and 60 eyes (39.2%) had a honeycomb appearance of different areas on the fundus. The supratemporal quadrant was the most commonly affected (45 eyes, 75.0%), followed by the infratemporal (23 eyes, 38.3%), the infranasal (10 eyes,16.7%), and supranasal (9 eyes,15.0%). The appearance was significantly associated with peripheral retinoschisis, inner retinal layer break, outer retinal layer break, RD, and rhegmatogenous retinal detachment (RRD) (*p* < 0.01, *p* = 0.032, *p* < 0.01, *p* = 0.008, *p* < 0.01, respectively). All the eyes complicated with RRD had the appearance. None of the eyes without the appearance had RRD.

**Conclusions:**

The data suggest that the honeycomb appearance is not uncommon in patients with XLRS and is more likely to be accompanied by an RRD, and inner and outer layer breaks, thus should be treated with caution and close observation.

## Background

X-linked retinoschisis (XLRS) is an inherited early-onset retinopathy, with an estimated prevalence between 1/5000 to 1/20000. It is caused by mutations in the RS1 (Gene ID: 6247; OMIM: 300839) gene in Xp22.1, which encodes a 224 amino acid protein, retinoschisin [1, 2]. The exact mechanism between protein abnormality in retinoschisin and the consequent photoreceptor degeneration is still unclear [3]. XLRS is characterized by a typical spoke-wheel macular schisis. Approximately 50% of the patients present peripheral retinoschisis [4]. Retinal detachment (RD) and vitreous hemorrhage (VH) are the most common complications and can cause severe deterioration of visual acuity [4–7]. Thus, the determination of risk factors for complications is crucial in evaluating a patient’s prognosis. Studies have shown that peripheral retinoschisis and bridging vessels are risk factors for RD and VH [8, 9]. Retinal honeycomb appearance is characterized by an irregularly excavated pockmarked appearance on the retina which can be seen on the fundus of XLRS patients. A similar appearance has been reported in patients with degenerative retinoschisis [10, 11] and is associated with a higher incidence of outer retinal break and rhegmatogenous retinal detachment (RRD) [11, 12]. However, its role in patients with XLRS has not been studied. We hypothesize that retinal honeycomb appearance is also associated with a higher incidence of outer retinal break and RRD in the setting of XLRS. This question was addressed with a retrospective chart review of 78 patients with XLRS. To our knowledge, this is the first report regarding the frequency and location of honeycomb appearance, and its association with complications in a large cohort of patients with XLRS.

## Materials and methods

This was a retrospective review of medical records and wide-field fundus imaging of all the patients diagnosed with XLRS at Beijing Tongren eye center between Dec 2017 and Feb 2022. All patients were initially diagnosed with XLRS by one of the authors (HL, JHL, SFL, YM) based on their clinical findings, such as decreased visual acuity, characteristic macular and/or peripheral retinoschisis and optic coherence tomography (OCT) findings, with or without a family history. Typical OCT findings included retinal splitting in the macular and/or peripheral area. Peripheral retinoschisis is the internal splitting of the peripheral retina [4]. Part of the patients was confirmed by the detection of a pathologic RS1 gene. We excluded patients with fundus changes other than those caused by XLRS, and patients that lack wide-field fundus imaging or adequate medical records, such as best corrected visual acuity (BCVA), intraocular pressure, slit-lamp examination, dilated binocular indirect fundus examination, and OCT. The data collecting and screening were done by JM, GDD, LL, and MZY. After reviewing 145 charts of all XLRS patients seen by one of the authors, 78 patients (153 eyes) were included.

Wide-field fundus photography was performed with the Optomap ultra-wide-field scanning laser ophthalmoscope 200 TX (Optos, Dunfermline, Scotland, United Kingdom) and/or the RetCam III digital camera (Clarity Medical Systems, USA). OCT was performed with the Spectralis OCT (Heidelberg Engineering, Heidelberg, Germany).

**Fig. 1:**
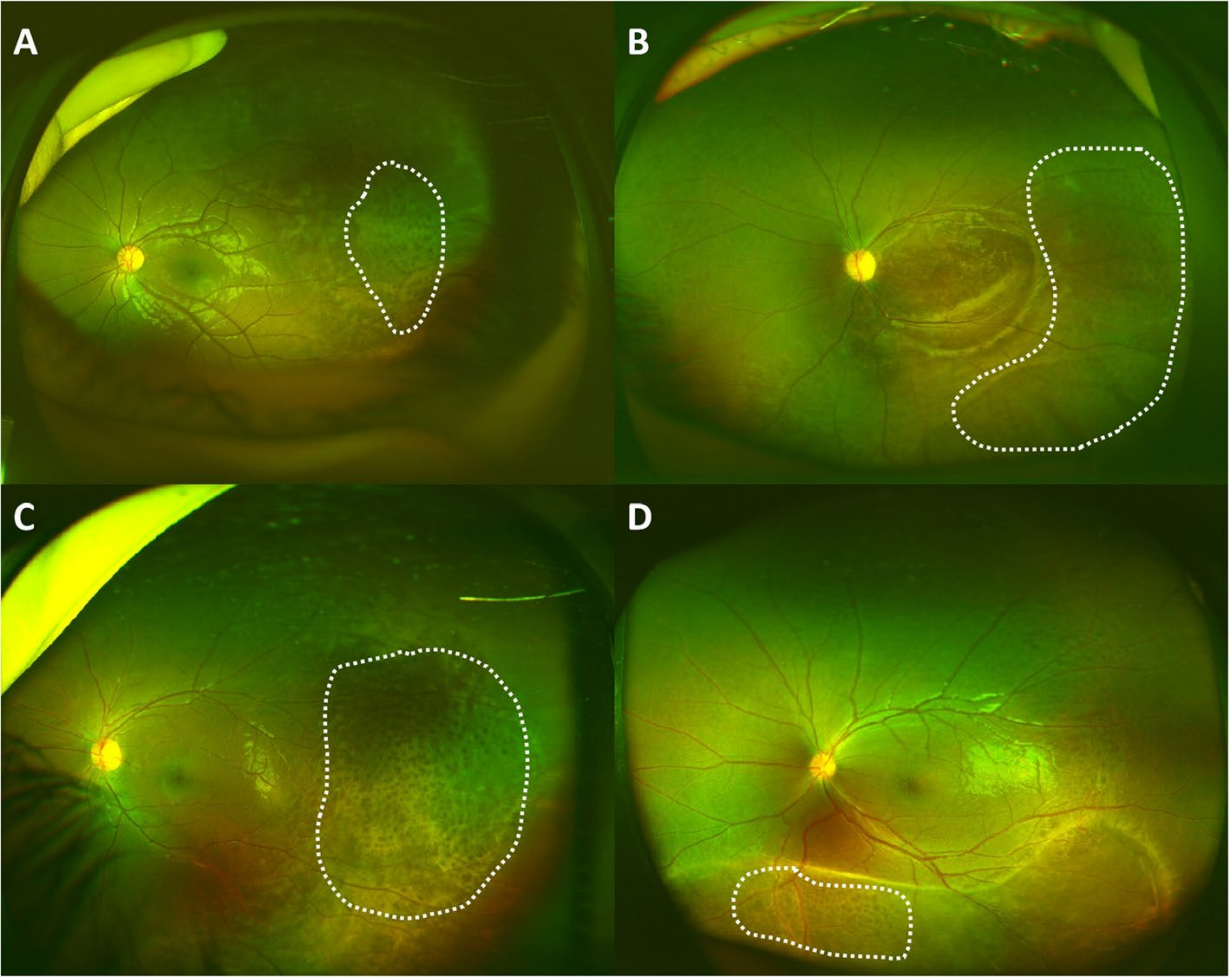
**A**-**D** Representative ultra-wide-field fundus photos depicting the honeycomb appearance of different areas in patients with XLRS (in the white dotted area)

**Fig. 2:**
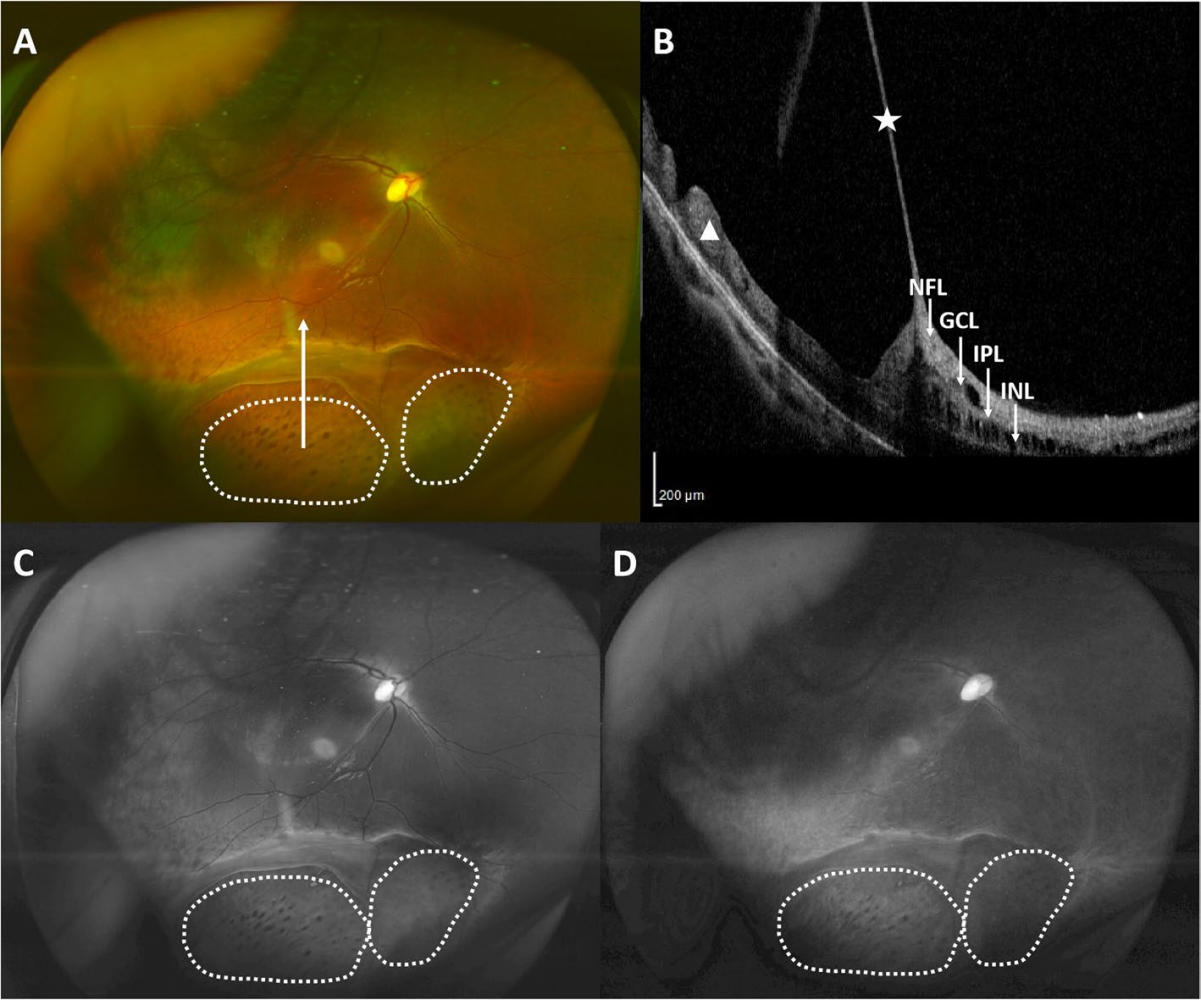
Representative ultra-wide-field fundus photos and OCT depicting retinal honeycomb appearance (in the white dotted area) in a patient with XLRS. **A** Retinal honeycomb appearance in the inferior quadrant in the right eye of a 5-year-old patient. The white arrowhead shows the scanning line of the OCT in **B**. **B** OCT scan showing splitting in the nerve fiber layer (NFL), ganglion cell layer (GCC) and inner nuclear layer (INL), a thin inner retinal layer (white star) and a relatively thick, excavated outer layer (white triangle). **C**, **D** The green channel (**C**) and red channel (**D**) of fundus photo 2A both showed an obvious honeycomb appearance, suggesting the excavation should involve a deeper retina

We defined retinal honeycomb appearance as an irregularly excavated pockmarked appearance on the whitish retina, with an area at least 1.75 mm in diameter (one average disk diameter), seen on the wide-field fundus photo. Representative fundus photos and OCT of the retinal honeycomb appearance are shown in Figs. 1 and 2.

### Statistical analysis

Statistical analyses were performed using SPSS Statistics 26.0 (IBM, Chicago, Illinois, USA). Mean ± SD and median (range) were used for describing continuous parameters. Numbers and percentages were used in categorical parameters. T-test was used for data with normal distribution and equal variances. The chi-square test or Fisher exact test was performed on the 2 × 2 cross-tabulations of honeycomb appearance and other peripheral retinal findings and complications. Statistical significance was defined as a *p* value < 0.05.

## Results

### Demographic and clinical features

Seventy-eight patients (153 eyes) with adequate clinical records and wide-field images were included. The patients were all male, and ranged in age from 2 to 26 years old, with a median age of 5.0 (4.0,7.0) years old. The logarithm of the minimal angle of resolution (logMAR) of BCVA was 0.19 ± 0.16, ranging from no light perception to 0.15. Twenty-two patients were confirmed by the detection of a pathologic RS1 gene, of which 17 were missense, 2 were small deletions, 2 were gross deletions and 1 was frameshift.

Regarding the macular change, 1 eye was normal (0.7%), 2 eyes showed epiretinal membrane (1.4%), 7 eyes showed atrophy (4.7%), 12 eyes were affected by an RD of any kind (8.1%), of which the number of eyes affected by RRD, tractional retinal detachment (TRD) and exudative retinal detachment (ERD) were 6, 6 and 0, respectively. Five eyes could not be judged due to the covering of a bullous retinoschisis, and 128 eyes (86.5%) showed macular schisis of different degrees. The frequencies and percentages of eyes with peripheral retinoschisis, inner retinal layer breaks, outer retinal layer breaks, complications, VH, RD, RRD, and TRD were 120 (78.4%), 83 (54.2%), 8 (5.2%), 55 (35.9%), 32 (20.9%), 34 (22.2%), 10 (6.5%) and 24 (15.7%), respectively (Table 1).

**Table 1:** Association between honeycomb appearance and complications and other peripheral findings

Honeycomb appearance						
		No	Yes	Total	χ2	*p* Value
	Total	93	60	153		
**Peripheral schisis**	No	33 (35.5%)	0(0.0%)	33(21.6%)	27.145	**<0.001**^**b**^
	Yes	60 (64.5%)	60(100.0%)	120(78.4%)		
**IRLB**	No	49 (52.7%)	21(35.0%)	70(45.8%)	4.597	**0.032**^**b**^
	Yes	44 (47.3%)	39(65.0%)	83(54.2%)		
**ORLB**	No	93 (100.0%)	52(86.7%)	145(94.8%)		**<0.001**^**a**^
	Yes	0 (0.0%)	8(13.3%)	8(5.2%)		
**Complications**	No	64 (68.8%)	34 (56.7%)	98(64.2%)	2.338	0.126^b^
	Yes	29 (31.2%)	26(43.3%)	55(35.9%)		
VH	No	74 (79.6%)	47(78.3%)	121(79.1%)	0.034	0.854^b^
	Yes	19 (20.4%)	13(21.7%)	32(20.9%)		
RD	No	79 (84.9%)	40 (22.2%)	119(77.8%)	7.051	**0.008**^**b**^
	Yes	14 (15.1%)	20(33.3%)	34(22.2%)		
RRD	No	93 (100.0%)	50(83.3%)	143(93.5%)		**<0.001**^**a**^
	Yes	0(0.0%)	10(16.7%)	10(6.5%)		
TRD	No	80(86.0%)	49(81.7%)	129(84.3%)	0.523	0.470^b^
	Yes	13(14.0%)	11(18.3%)	24(15.7%)		

Values are shown as the number of eyes (with the percentage of the total in parenthesis)

*IRLB* Inner retinal layer break, *ORLB* Outer retinal layer break, *VH* vitreous hemorrhage, *RD* retinal detachment, *RRD* rhegmatogenous retinal detachment, *TRD* tractional retinal detachment

Values in bold font are statistically significant at *p* < 0.05

^a^Fisher exact test

^b^ Chi-square test

### Clinical features of retinal honeycomb appearance

Thirty-eight patients (48.7%), and 60 eyes (39.2%) had a honeycomb appearance of different areas on the fundus. There was no significant difference in age at onset between patients with and without the honeycomb appearance (6 ± 4 VS 7 ± 5, t = − 0.8, *p* = 0.437). The BCVA of patients with the honeycomb appearance was worse than those without the appearance but of no significance (1.0 ± 0.4 VS 0.9 ± 0.6, t = 1.1, *p* = 0.276). Nine were in the right eye (9.0%), 7 were in the left (10.3%), and 22 were bilateral (28.2%). The supratemporal quadrant was the most commonly affected (45 eyes, 75.0%), followed by the infratemporal (23 eyes,38.3%), the infranasal (10 eyes,16.7%), and supranasal quadrant (9 eyes,15.0%). All the honeycomb appearances were found within the area of retinoschisis. Most of the appearance was found near the equator, and in 17 eyes (28.3%) extended to the posterior retina. OCT showed splitting in the more superficial level of the retinal, thus resulting in an extremely thin inner layer and a relatively thick outer layer. The outer layer was irregularly excavated to produce a honeycomb appearance. The honeycomb appearance can be seen in both the red and green channels of the ultra-wide-field retinal imaging system when available (Fig. 2).

### Relationship between the honeycomb appearance and other peripheral retinal findings

The appearance was significantly associated with peripheral retinoschisis, inner retinal layer break, and outer retinal layer break. All the honeycomb appearances were found within the area with retinoschisis (*p* < 0.01). Out of the 60 eyes with the honeycomb appearance, 39 (65.0%) had inner retinal layer breaks, 8 (13.3%) had outer retinal layer breaks, compared with only 44 (47.3%) had inner layer breaks and none had outer layer breaks in eyes without the appearance (*p* = 0.032, *p* < 0.01) (Table 1).

### Relationship between honeycomb appearance and complications

**Fig. 3:**
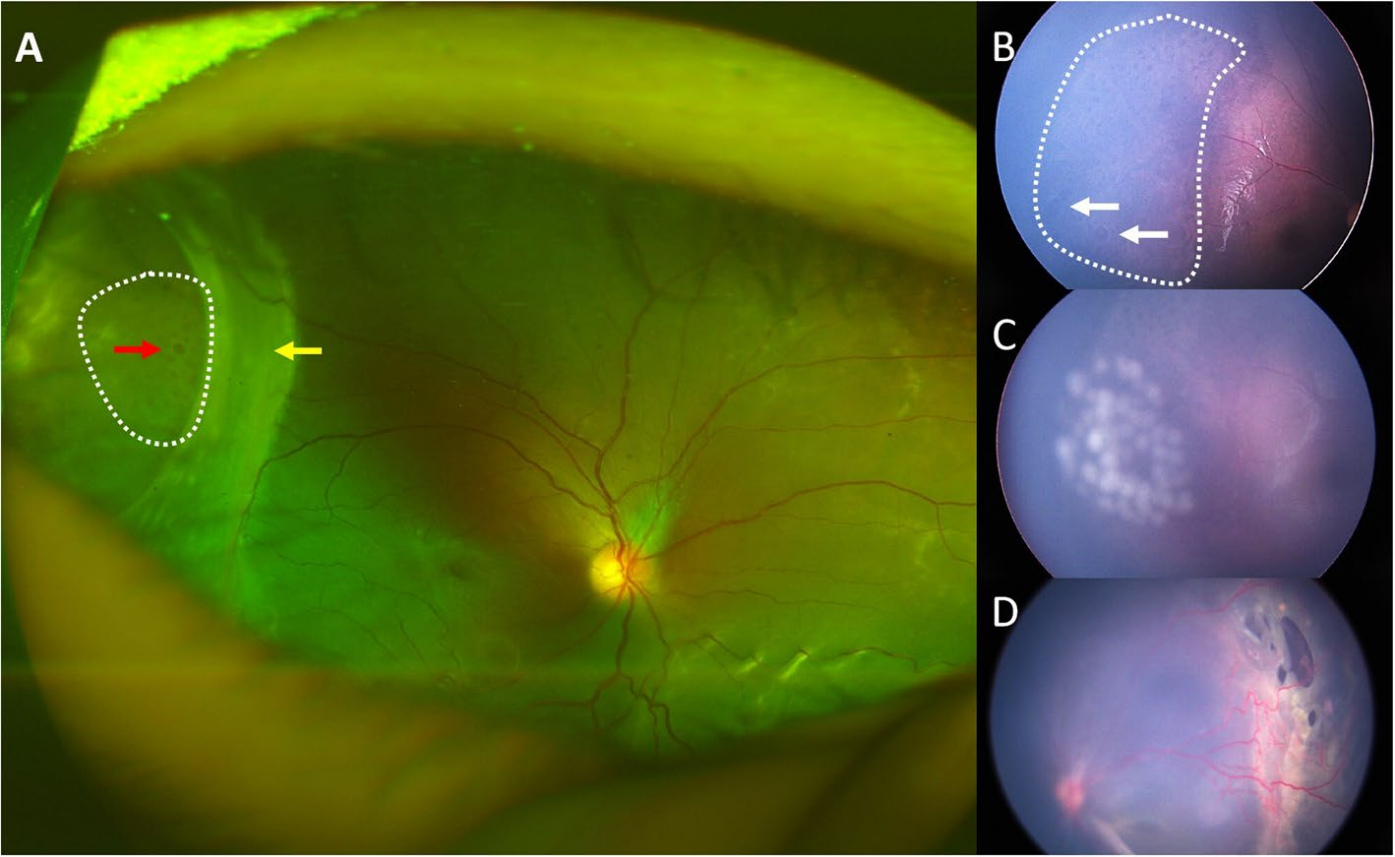
Representative fundus photos of the retinal honeycomb appearance accompanied by inner retinal layer break. **A** Retinal honeycomb appearance (area in the white dotted area) with a giant inner retinal break on the inner layer (yellow arrow) and outer retinal layer break (red arrow) in the temporal quadrant in the right eye with chronic RRD of a 3-year-old patient. **B**-**D** RetCam fundus photos of another 3-year-old patient. The patient had a typical honeycomb appearance (white dotted area) and multiple inner layer breaks in his right eye (white arrows) (**B**) and was treated with prophylactic laser photocoagulation (**C**). The outer retinal layer was still attached at the time of treatment, but the eye suffered an RRD later during follow-up. The left eye presented with a chronic RRD with multiple breaks in the temporal quadrant at onset (**D**), symmetrical with the area of inner layer breaks and the honeycomb appearance in the right eye

The occurrence of complications was determined by the medical record and fundus photo. The honeycomb appearance was significantly associated with RD and RRD (*p* = 0.008, *p* < 0.01). Out of the 34 eyes with RD, 20 (33.3%) had the appearance. All the eyes complicated with RRD had the honeycomb appearance (Table 1, Fig. 3). None of the eyes without the honeycomb appearance had RRD. One of the patients had a typical honeycomb appearance and multiple inner layer breaks in his right eye at the same time at onset and was treated with prophylactic laser photocoagulation, but still suffered an RRD during follow-up (Fig. 3B-C).

## Discussion

XLRS is a congenital retinopathy with variable natural history [5, 13]. The visual acuity of most patients remains relatively stable or gradually declines in childhood and may suffer further deterioration due to macular atrophy in adulthood [4], while others may suffer sudden severe deterioration at a younger age due to complications like RD and VH [13, 14]. Macular and peripheral retinoschisis are typical features of XLRS, with previous reports in the literature ranging from 68% to 100% and 43% ~ 60%, respectively [7, 15]. We report macular schisis in 86.5% and peripheral retinoschisis in 79.3% of the eyes in our cohort, which is relatively high, possibly due to the younger age of our patients, considering the macular and peripheral retinoschisis will collapse and change into atrophic manifestation later in life [13]. We report inner layer breaks in 55.2% and outer layer breaks in 5.3% of the eyes in our cohort, which is in accordance with the previous report of inner layer breaks in about 52% of the eyes and outer layer breaks being rare but without precise percentage [15]. We report RD in 21.6% of the eyes, compared with 5.5–16% in previous reports [9], and vitreous hemorrhage in 20.9% of the eyes, compared with 3–21% in previous reports [9].

We report, for the first time, the clinical characteristics of honeycomb appearance in the fundus of patients with XLRS. Retinal honeycomb appearance is defined as an irregularly excavated pockmarked appearance on the whitish retina [11], and was found in the wide-field fundus photo of 38 patients (48.7%), and 60 eyes (39.2%). OCT showed an extremely thin inner layer and an irregulated excavated outer layer. The Optomap ultra-wide-field fundus image is captured with two laser wavelengths: red channel at 633 nm and green channel at 532 nm that scan the retina simultaneously. The red channel permits a view of deeper retinal structures [16]. In our cohort, the honeycomb appearance could be seen in both the red and green channels, indicating the excavation could be in a rather deep level of the retina. The quick acquisition and wide-field nature of the Optomap fundus imaging system make it possible to get a more comprehensive view of the peripheral fundus of younger patients, which otherwise would be impossible without proper anesthesia. The fact that, in our cohort, most of the outpatient records failed to include a description of the honeycomb appearance, even when the appearance was rather obvious on the wide-field images, may explain the reason why this appearance may be underestimated in our clinical practice. Also, a lack of recognition of this unique appearance may contribute to this discrepancy. We usually concentrated more on the severity of the retinoschisis and whether there was RD or retinal breaks in the fundus examination before. With the recognition of this unique appearance on wide-field imaging and specific attention, we could easily find this appearance in the subsequent fundus examination.

The previous report regarding the honeycomb appearance is rare. But similar appearance has been reported in studies of degenerative retinoschisis. Mohite et al. report a bullous degenerative retinoschisis with a typical honeycomb appearance [10]. In a study based on 2319 eyes obtained in autopsy and 50 eyes with degenerative peripheral retinoschisis that were clinically examined, degenerative retinoschisis can be classified as typical or reticular. Typical retinoschisis presents a deeper splitting level, usually in the outer plexiform and inner nuclear layers, and is more likely to present in the pre-equatorial retina and does not usually extend into the posterior retina. It is rarely associated with laminar retinal breaks and retinal detachment. Reticular retinoschisis, a more severe type, has a more superficial splitting level, usually between the inner limiting membrane to the inner plexiform layer. Reticular retinoschisis has a predilection for the inferior temporal quadrant and is round or ovoid with a bullous elevation of the extremely thin inner layer and an irregular excavated outer layer thus presenting a honeycomb appearance. Reticular retinoschisis often extends into the posterior retina and is frequently associated with outer layer breaks (23–25%) and is more likely to be complicated with a retinal detachment (up to 22%) [11, 12].

In our cohort, the honeycomb appearance exhibited similar clinical characteristics to the reticular retinoschisis. All the honeycomb appearances were found within the area of retinoschisis. The splitting level was also superficial and showed excavated outer layer in the OCT, in accordance with the pathological change of the reticular retinoschisis. Previous histopathological studies of XLRS show that peripheral schisis occurs mainly in the superficial layers, such as the nerve fiber layer and ganglion cell layer [17–19]. OCT-based studies show that schisis can present in various layers, with different regional distributions [20–22]. Jia Yu et al. report that schisis of the inner nuclear layer, outer nuclear layer, and outer plexiform layer almost always involves the foveal center, but the schisis of the retinal nerve fiber layer is seen only in the parafoveal area [23]. Han et al. report the schisis of the ganglion cell layer to be perifoveal and the schisis of the outer nuclear layer to be mostly extramacular [20]. However, little is known regarding the schisis distribution in the far periphery region. The difference in the regional distribution of schisis may be explained by the difference in the retina structure between the macular and the periphery. The density of nerve fibers entering the nerve fiber layer is greater in the posterior pole, thus a stronger force, or disease at a more advanced stage, may be required to separate the ganglion cell layer from the retinal nerve fiber layer in this area to create schisis [23].

Regarding complications, we report here that the honeycomb appearance was significantly associated with peripheral retinoschisis, inner and outer layer breaks, RD and RRD. The relatively thin inner layer may make itself more vulnerable and susceptible to an inner layer break. In our cohort, there was a patient who demonstrated a typical honeycomb appearance and inner retinal break at the same time. Despite a prophylactic laser photocoagulation treatment, the affected eye still suffered an RRD during follow-up. We propose that honeycomb appearance may be a precursor of an outer layer break, considering with the progression of the disease, the excavation in the outer layer may coalesce into a full-thickness break, that is, an outer layer break. Concurrent outer and inner layer breaks are risk factors for RRD. And this may explain the higher incidence of outer retinal break and RRD in eyes with a honeycomb appearance. Hence, eyes with this specific appearance warrant closer follow-up and maybe more proactive prophylactic treatment. However, more cases and longer follow-ups are needed to investigate the natural course of the eyes with a honeycomb appearance and inner retinal breaks at the same time and to evaluate the safety and effectiveness of prophylactic laser treatment or any other possible treatment.

We acknowledge several limitations to this study. The retrospective nature of the design lacks a longitudinal follow-up to further investigate the change of the honeycomb appearance. Due to the relatively young age and the subsequent lack of cooperation of this cohort, a satisfying acquisition of the peripheral fundus image and OCT scans was difficult. Most of the OCT scans were of the macular area. Further OCT-based study regarding the periphery area of XLRS, especially the area with a honeycomb appearance is warranted, to provide more insight into this appearance. Also, the number of patients with genetic data in our cohort is too small to be analyzed. A genotype-phenotype correlation study in the future would give further insights into the disease pathology and this retinal appearance.

## Conclusion

This study reports the clinical characteristics of honeycomb appearance in a large cohort of XLRS patients for the first time. The data suggest that honeycomb appearance is not uncommon in patients with XLRS, and is more likely to be accompanied by inner and outer layer breaks and RRD, thus should be treated with caution and close observation.

## Data Availability

All data generated or analyzed in this study are included in this published article.

## Abbreviations

XLRS: X-linked retinoschisis
OCT: Optical coherence tomography
BCVA: Best corrected visual acuity
logMAR: The logarithm of the minimal angle of resolution
NFL: The nerve fiber layer
GCC: Ganglion cell layer
INL: Inner nuclear layer
IRLB: Inner retinal layer break
ORLB: Outer retinal layer break
VH: Vitreous hemorrhage
RD: Retinal detachment
RRD: Rhegmatogenous retinal detachment
TRD: Tractional retinal detachment
